# Up close and personal: poverty and human development

**Published:** 2007-10-22

**Authors:** Sally Murray, James Brophy, Claire Kendall, Anita Palepu

## Abstract

Poverty and health are inextricably linked: poverty diminishes access to health care (whether through reduced ability to pay, a lack of knowledge about when to seek health care or a lack of adequate services within reach), increases exposure to disease and other illness (for example, through exposure to dangerous workplaces), and is related to reduced access to clean water, housing and sanitation.

October 22 marks the launch of special issues related to global poverty and human development in more than 200 biomedical journals around the world.* Although poverty contributes to poor health in more than 2 billion people worldwide, and despite an exponential increase in scientific research publication in recent years, little is published on interventions and analyses that target improved health in poor countries. *Open Medicine*’s participation in this collaborative initiative is a small effort toward redressing this deficiency.

Poverty and health are inextricably linked: poverty diminishes access to health care (whether through reduced ability to pay, a lack of knowledge about when to seek health care or a lack of adequate services within reach), increases exposure to disease and other illness (for example, through exposure to dangerous workplaces), and is related to reduced access to clean water, housing and sanitation.

The need to address poverty as an underlying contributor to poor health is not a new concept: the 1978 Alma-Ata Declaration determined that achieving the highest possible level of health requires the response not only of the health sector but of many other social and economic sectors as well.^1^ Yet it is only in the last decade that agencies such as the World Health Organization have focused on poverty as a distinct determinant of health.^2^, ^3^

Other initiatives such as the United Nations Millennium Development Goals (MDGs) similarly have poverty as a focus.^4^ The MDGs focus on reducing poverty and hunger as well as addressing specific health issues (*e.g.*, maternal health, child mortality, HIV/AIDS and malaria) and broader, interrelated issues such as education, gender equality and environmental sustainability. The initiative brings sharp focus to the problem of poverty as a determinant of health and of human development.

Arguably, some agencies may have pursued the link between poverty and health less out of an interest in health as a fundamental human right (as argued at Alma-Ata) than through the realization that improved health leads to economic gain and gross domestic product (GDP) growth. Importantly — irrespective of the underlying reasons for addressing the problem — this change in direction in multi-agency attention has focused much-needed resources on neglected health sectors and initiatives addressing poverty.

As we proceed to address poverty and human development we need to ensure that our work is done with care. The way we calculate, benchmark and monitor improvements (or deterioration) across countries and continents can hide as much as it reveals. Using aggregate statistics may in fact silence some underprivileged groups whose health (or other) outcomes are subsumed by the statistics generated by larger population groups. For example, small groups within countries may, as a result of poverty, lack opportunity for human development and not be adequately represented in national statistics.

The Indigenous people of Australia provide one example of the multifaceted impact of poverty on health. Australia is home to 21 million people, including 410,000 Indigenous Australians.^5^ Although the incomes of Indigenous Australians’ do not meet the United Nation’s criteria for absolute poverty (less than US$1 per day) the income gap in Australia means that the problems plaguing the world’s poor are mirrored in these communities, in reduced life expectancy, low schooling rates, poor access to water and sanitation, and reduced access to health care.

The extent of the disparity between Australian national averages and those of Indigenous Australians can be highlighted using the UN Human Development Index (HDI). The HDI draws attention to three facets of human development — life expectancy, education and standard of living — and is used to make comparisons at the country level.^6^ Australia is ranked third in the index. If we examine these three indicators for Indigenous Australians we see that Australia’s ranking hides the experiences of Indigenous Australians.

Consider life expectancy and education. Indigenous Australians can expect to live 17 years less than non-Indigenous Australians (59 years for Indigenous men), meaning that life expectancy in Nigeria, Nepal, India and Bangladesh is considerably greater.^6^ Schooling rates in Australian Indigenous communities are also considerably lower. Indigenous persons are only half as likely as non-Indigenous persons to have completed Year 12 (18% compared with 41%) and only one-quarter as likely if they are living in remote areas.^5^, ^7^ Saliently, a 2003 review of schooling in Indigenous communities found that poverty limits literacy and numeracy.^8^

The third ingredient of the HDI—standard of living— is also clearly substandard. The HDI uses purchasing power parity (PPP) and income to measure standard of living. Although PPP data are not available for Indigenous Australians, raw income data show that for the period 2002 to 2004–05 the median gross weekly household income for Indigenous Australians was A$340, almost half that of non-Indigenous population (A$618), and that 40% of Indigenous people had household incomes in the lowest quintile.^5^

Receiving an income is, at least in part, dependent on participation in paid employment. In 2004–05 over half of Indigenous people received their individual income from government pensions and allowances, and a further 10% received income from government work schemes.^5^ Today, fewer than half of Indigenous people aged 15 years or more report paid employment (42%), and Indigenous people are almost three times more likely than non-Indigenous people to be unemployed (20% compared with 7%).^7^

Indigenous Australians’ reduced income (and potential access to income) is compounded in other ways: up to 85% of income is spent on basic living costs in remote communities, with food accounting for almost one third of the total^9^ (cited in Webb and Leeder).^10^ This compares with Australian population spending of less than 20% of income on food.^11^ Access to health services is often limited, housing is unavailable or unaffordable, and basic services such as sanitation and water are still, astoundingly, unavailable in some communities.

The experience of Indigenous Australians is not dissimilar to that of Indigenous peoples around the world. In every instance, Indigenous peoples of the world have life expectancies lower than the national average.^3^ Their levels of income, education and employment are also worse. In Canada life expectancy for First Nation peoples is 7.4 years (males) and 5.2 years (females) less than the national average.^12^ Educational attainment indicators, including secondary school completion rates, postsecondary education admissions and completion of university degrees are lower for on-reserve Registered Indians^12^ and, like Indigenous Australians, most Aboriginal people are at or below the poverty line. In major Western Canadian cities, four times as many Aboriginal people as other citizens live below the poverty line.^13^

The Commission on Social Determinants of Health (CSDH), a World Health Organization-affiliated body, recognizes the unique problems facing Indigenous peoples across the globe and are committed to specifically examining the health of these populations. The Commission held a recent symposium on Indigenous health in Adelaide, Australia, for 74 representatives from Australia, Belize, Cambodia, Canada, Chile, China, Ecuador, Guatemala, New Zealand, the Philippines and the United Kingdom.

The report from the Symposium underlines the resolution of Indigenous poverty as “fundamental to improving health.” Income, education and employment are highlighted for their interdependent impact on health, each leading to “marginalisation [and] limiting access to education, employment, good housing and nutritious food.” Representatives also found that poverty has a direct impact on mental well-being by lowering self-esteem, increasing dependence and “vitiating one’s ability to participate fully in society.”^14^

The Symposium’s report highlights investment in education, particularly of children, as one means of combating poverty. Given the impact of education on subsequent income, employment status and living conditions, this is a crucial part of action^3^ and one also supported by the MDGs.^4^

In Australia there are growing (but belated) efforts to do just this. A recent government report highlights several initiatives that are successfully encouraging Indigenous children’s education. These include Deadly Vibe, a magazine for Indigenous students published by an Aboriginal media agency, non-government-sector sponsorship of scholarship programs for children to board at private schools, and a private foundation engaging and supporting young Indigenous men to complete Year 12 and to find employment.^5^ Unfortunately, such initiatives are unlikely to be replicated in poorer nations with few private benefactors.

Poverty is a complex phenomenon, no less in countries with Indigenous people, where poverty is tied to the limitation of opportunity through racism, geographical isolation and political inertia. As fraught and complex as the solutions may be, there is no doubt that broader global efforts must be cognisant of Indigenous poverty and opportunity. In the same way, we must consider that Indigenous poverty is just one example of poverty in the context of affluence. Many countries have broad income gradients and large gaps between the very rich and the very poor.^15^ We must ensure that our efforts address all groups in need.

We hope that the research, commentary and analysis published this month in *Open Medicine* and the other many journals offer useful debate and information for policy-makers, health practitioners and program managers with an interest in global poverty and human development. The articles *Open Medicine* publishes are open access, freely accessible to all. We believe there is little point in publishing key information to influence health outcomes and debate if those who want to use the information are impeded from accessing it by expensive subscription fees. Indeed, open access publication of scientific and technical information is supported by the UN as part of building a people-centred, inclusive and development-oriented society.^16^† We also hope that research is used carefully and critically; ensuring that all are included in our poverty reduction mandate to support true human development.

## Footnotes

* See a full list of participating journals.

† The WSIS principles and plan of action are endorsed by the UN General Assembly resolution 59/220.
